# Single-Step Genomic Evaluation for Meat Quality Traits, Sensory Characteristics, and Fatty-Acid Composition in Duroc Pigs

**DOI:** 10.3390/genes11091062

**Published:** 2020-09-09

**Authors:** Bryan Irvine Lopez, Kier Gumangan Santiago, Donghui Lee, Younggyu Cho, Dajeong Lim, Kangseok Seo

**Affiliations:** 1 Division of Animal Genomics and Bioinformatics, National Institute of Animal Science, Rural Development Administration, Wanju 55365, Korea; irvinelopez@korea.kr (B.I.L.); lim.dj@korea.kr (D.L.); 2 Department of Animal Science and Technology, Sunchon National University, Suncheon 57922, Korea; santiagokier2015@gmail.com (K.G.S.); a3832737@naver.com (D.L.); dudrb1992@naver.com (Y.C.); 3 Department of Animal Science, College of Agriculture, Central Luzon State University, Science City of Muñoz 3120, Philippines

**Keywords:** single-step genomic BLUP, meat quality, sensory characteristics, fatty acids

## Abstract

Meat quality and carcass characteristics have gained the attention of breeders due to their increasing economic value. Thus, this study investigated the genomic prediction efficiencies of genomic best linear unbiased prediction (GBLUP) and single-step GBLUP (ssGBLUP) for traits associated with meat quality, sensory characteristics, and fatty-acid composition. A total of 1237 Duroc finishing pigs with 654 individuals genotyped using the Illumina Porcine SNP 60k marker panel were used in this study. Prediction accuracy and bias for GBLUP and ssGBLUP were evaluated using a five-replicates of five-fold cross-validation. Estimation of genetic parameters for traits associated with meat quality, including lightness, yellowness, redness, pH at 24 h post-mortem, moisture content, fat content, water-holding capacity, cooking loss except for shear force (0.19), as well as fatty-acid composition (palmitic, stearic, oleic, linoleic, and linolenic fatty acids), revealed moderate to high heritability estimates ranging from 0.25 to 0.72 and 0.27 to 0.50, respectively, whereas all traits related to sensory characteristics (color, flavor, tenderness, juiciness, and palatability) showed low heritability estimates ranging from 0.08 to 0.14. Meanwhile, assessment of genomic prediction accuracy revealed that ssGBLUP exhibited higher prediction accuracy than GBLUP for meat quality traits, fatty-acid composition, and sensory characteristics, with percentage improvements ranging from 1.90% to 56.07%, 0.73% to 23.21%, and 0.88% to 11.85%, respectively. In terms of prediction bias, ssGBLUP showed less bias estimates than GBLUP for the majority of traits related to meat quality traits, sensory characteristics, and fatty-acid composition of Duroc meat. In this study, ssGBLUP outperformed GBLUP in terms of prediction accuracy and bias for the majority of traits. Through selection and breeding, our findings could be used to promote meat production with improved nutritional value.

________________________________________________________________________________________

## 1. Introduction

Pork has been an important human food for thousands of years [1]. Globally, a four-fold increase in pork production has been observed over the past five decades, and this is expected to continuously increase over the next three decades [2]. From 1970 to 2012, South Korea showed a continuous increase in the consumption of pork, beef, and chicken, fruits, and vegetables while the consumption of rice and barley decreased [3]. Related to this, the USDA-Foreign Agriculture Service [4] reported that South Korea ranks as the second and fifth-largest (by value) market for US-produced beef and pork, respectively. This increase in pork importation of US red meat by South Korea can be attributed to the country’s growing economy, consumers’ preferences, and the lack of arable land for livestock feed production [4]. Moreover, according to the Korea Rural Economic Institute (KREI, 2015) [5], pork importation necessarily increased due to a shortage of pork production versus consumer demand, which resulted in a decrease in the self-sufficiency rate of the country from 92.40% in 1990 to 81.10% in 2013. In terms of consumption, Koreans show lower preferences for carcass parts with low-fat content; instead, they show high demand for pork belly and Boston butt [6], indicating strong preferences for pork high in fat and marbling.

According to Choi et al. [7], the majority of pork found in the Korean market is sourced from three-way crossbred pigs (Landrace × Yorkshire Dam × Duroc Sire, LYD), known for having high litter size, faster growth performance, and a higher volume of meat production. In a study by Kim et al. [8], Yorkshire (Y) and Landrace (L) pigs were observed producing a higher volume of retail cut (by weight) and longer carcass length than Duroc (D) pigs, respectively. However, in terms of meat quality characteristics, Choi et al. [9] revealed that purebred Duroc possesses better meat quality characteristics, higher intramuscular fat and pH values, better flavor, and juiciness, and lower drip loss and cooking loss than three-way crossbred pigs (LYD). This observation suggests that a higher bloodline of Duroc (50%) in LYD crossbred pigs is important in satisfying consumer marbling preferences, although Landrace and Yorkshire are equally important due to their benefits of increasing carcass yield and maximizing the economic profitability of the fattening operation.

In recent years, swine breeding objectives have been extended to include pork quality and carcass characteristics due to their economic value [10]. However, in the past, genomic prediction of pork quality using traditional best linear unbiased prediction (BLUP) and indirect selection methods based on correlated traits was a sluggish process, expensive to measure, and required post-mortem examinations [11]. According to Goddard and Hayes [12], a previous approach based on single nucleotide polymorphisms (SNPs) and statistical methods that efficiently capture the effects of SNPs resulted in a two-fold higher genetic improvement rate per annum in many livestock systems. Among the commonly used statistical methods that require genotype data are genomic best linear unbiased prediction (GBLUP) and single-step GBLUP (ssGBLUP). In a study by Christensen et al. [13], the genomic prediction accuracy of the single-step method (ssGBLUP) outperformed multi-step methods like GBLUP. Therefore, the objectives of this study are (1) to compare the accuracies and unbiasedness of predicted genomic breeding values obtained using the GBLUP and ssGBLUP methods for traits related to meat quality, sensory characteristics, and fatty-acid composition of Duroc meat and (2) to determine the additive genetic, residual, and phenotypic variances as well as heritability estimates of all traits included in this study.

## 2. Materials and Methods

### 2.1. Animals

A total of 1237 female Duroc finishing pigs were used as a source of meat samples in this study. These pigs were initially reared from the period 2012 to 2018 in five breeding farms under the genetic improvement program of the South Korean government. Briefly, pigs were delivered and raised under commercial finishing conditions with open-front finisher building, partially slotted floor, and pen space per pig of 1.4 m^2^. In terms of genetic relatedness, a high connectedness rating between Duroc breeding farms was presented in our previous study [14]. Such high connectedness rating was expected, since these breeding farms participated in the semen exchange program.

### 2.2. Phenotypes

The initial records for each pig include the following information: pedigree, registration number, birth date, parity number, sex, birth weight, and end weight. All pigs that reached the average slaughter age and weight of 5 to 6 months and 97 kg, respectively, were delivered and slaughtered in commercial slaughterhouses following the Korea Institute for Animal Production Quality Evaluation (KAPE) standards. Meat samples were collected from the slaughterhouses and utilized as the experimental material for the measurement of meat quality traits, including lightness, redness, and yellowness of meat, pH level at 24 h post-mortem, moisture and fat content, water-holding capacity, cooking loss, and shear force; sensory characteristics such as color, flavor, tenderness, juiciness, and palatability; and fatty-acid composition, including palmitic (C16:0), stearic (C18:0), oleic (C18:1), linoleic (C18:2), and linolenic (C18:3) fatty acids. The approval of the Animal Care and Use Committee was not necessary for this study since initial records were acquired from the existing database and no animal experimentation was conducted as all meat samples used in this study came from the slaughterhouse.

### 2.3. Meat Quality Measurements

The collected meat samples were immediately delivered to the laboratory for the measurement of meat quality traits within 24-h post-slaughter. The meat color for each sample was expressed into three coordinates: lightness (L* = 100 is completely white, L* = 0 is completely black), redness (positive a* value is red while negative a* value is green), and yellowness (positive b* is yellow while negative b* is blue) based on the color space defined by the International Commission on Illumination (CIE L*a*b*). The average values for each coordinate were measured after cutting and blooming the meat for 15 min using a Konica Minolta Spectrophotometer CM-2500d equipped with an 8 mm measuring port, D65 illuminant, and 10° standard observer. The pH value of meat samples at 24-h post-mortem was measured using an Orion 3 Star (Thermo Electron Corporation, Waltham, MA, USA). The moisture content (MC) was measured by following the AOAC loss on drying method (AOAC, 1995) [15], in which 2 g of loin per meat sample is oven-dried for 104 °C and subsequently air-dried for 24 h before the determination of percent moisture content using a mathematical equation. Fat content (FC) in this study was extracted from externally trimmed fat loin muscle using organic solvent following the method described by Folch et al. [16].

The water-holding capacity (WHC) was determined after ground meat was placed on a centrifugation tube and centrifuged at a speed of 1000 rpm for 10 min. Cooking loss was measured after cooking the standardized loin samples for 2 min and reaching an internal temperature of 72 °C using a double-sided electric meat grill (Nova EMG-533, 1400 W, Evergreen Enterprise, Yongin, Korea). Shear force was measured by cutting loin (cooked meat) with longitudinal muscle fiber oriented perpendicularly using a Warner–Bratzler shear attachment on a texture analyzer (TA-XT2, Stable Micro System Ltd., Surrey, U.K.).

### 2.4. Sensory Evaluation

The sensory evaluation for color, flavor, tenderness, juiciness, and palatability of Duroc meat was done by cooking meat samples in an electric meat grill (Nova EMG-533, 1400 W, Evergreen Enterprise, Yongin, Korea) until reaching an internal temperature of 75 °C. Cooked meat samples were then placed in coded dishes and were judged by trained panelists based on a 9-point hedonic scale (1 = very bad to 9 = very good). The average score given by the trained panelists for each sensory parameter was subsequently used in the formal analysis.

### 2.5. Fatty-Acid Composition

The data for fatty acids were assessed from sample tissues of loin muscle immediately collected after slaughter and kept frozen by storage at −20 °C. The total lipid content from meat samples used in this study was quantified by following the protocol from previous studies based on the methodology presented by Folch et al. [16]. Concisely, the saponification of extracted fats was done using potassium hydrate-ethanol solution and methyl esterification was done using boron trifluoride-methanol solution. The quantification of fatty acids was done by using gas chromatography (Model 6890, Hewlett-Packard Co., Wilmington, DE, USA).

### 2.6. Genotyping and Genomic Quality Control

A total of 654 finishing pigs were genotyped for 61,564 SNPs using an Illumina Porcine SNP60K (Illumina, Seoul, South Korea) bead chip according to the manufacturer’s protocol. All genotypes were then subjected to quality control using PLINK v. 1.9 [17]. All SNPs with call rates of lower than 0.90, minor allele frequencies lower than 0.01, and Hardy–Weinberg disequilibrium (HWE) with a *P*-value less than 10^−7^ were all removed as part of the genotype data. After quality control, only 645 Duroc pigs and 34,297 SNPs were retained for subsequent analysis.

### 2.7. Derivation of Corrected Phenotypes

Corrected phenotypes (*y***_c_**) were used as response variables in the genomic prediction analyses. Here, *y***_c_** was calculated by adding pedigree-based estimated breeding values (EBV) to estimated residuals for each trait. The model used to estimate EBV was (Equation (1)):

(1) y=Xb+Za+e

where *y* is the vector of observations for each trait; b is the vector for all fixed effects, including herd (5 levels), slaughter year (8 levels), slaughter month (12 levels), and a covariate of slaughter weight (meat quality) or fat contents (fatty acid traits); a is the vector of additive genetic effects; e is the vector of random residual effects; and X and Z are the indices matrices for fixed and additive genetic effects, respectively. Meanwhile, a total of 4498 animals with 991 sires and 2288 dams were traced back to construct a pedigree relationship matrix.

### 2.8. Genomic Prediction

The GBLUP method based on the genomic relationship matrix as well as the ssGBLUP method with a joint relationship matrix constructed from marker and pedigree information were used to predict breeding values. A single-trait analysis was used for all traits.

#### 2.8.1. Genomic Best Linear Unbiased Prediction (GBLUP)

The GBLUP model was used to estimate the genetic merit of all genotyped individuals (Equation (2)):

(2) $$y_{c}=1\mu +Zg+e$$

where *y_c_* indicates the vector of corrected phenotypes of genotyped animals; μ signifies the overall mean; 1 denotes the vector of ones; *g* is the vector of genomic estimated breeding value (GEBV) obtained using GBLUP following the normal distribution $N\left(0,\;G\sigma_{g}^{2}\right)$, where $\sigma_{g}^{2}\;$ is the additive genetic variance and G is the marker-based relationship matrix [18]; e is the vector of random errors following the normal distribution of $N\left(0,\;I\sigma_{e}^{2}\right)$, with $\sigma_{e}^{2}$ indicating residual variance; I is the identity matrix; and Z is the incidence matrix that links *g* to *y_c_*. According to Van Raden [18], the G matrix was created as follows (Equation (3)):

(3) $$G=\frac{\left(M-P\right){\left(M-P\right)}^{\prime }}{\sum_{j=1}^{m}2p_{j}\left(1-p_{j}\right)}$$

where M represents matrix genotypes; P is a matrix containing the frequency of the second allele (p_j_), expressed as 2p_j_; *m* indicates the SNP numbers; and p_j_ signifies frequency of an allele *j-th* SNP.

#### 2.8.2. Single-Step Genomic Best Linear Unbiased Prediction (ssGBLUP)

The single-step GBLUP (ssGBLUP) method uses a combined relationship matrix (H) that integrates genomically derived relationships (G) and pedigree-based relationships (A), which allows a one-step procedure for genomic selection [19]. The only difference between GBLUP and ssGBLUP is that the H matrix used by the latter method replaces G used in the GBLUP method. The inverse form of the H matrix was presented in the simplest form by Legarra et al. [20] and Aguilar et al. [21] (Equation (4)):

(4) $$H^{-1}=A^{-1}+\left[\begin{array}{c} 0\\ 0 \end{array}\;\begin{array}{c} 0\\ {\;G}^{-1}-A_{22}^{-1} \end{array}\right]$$

where A is the inverse of the numerator relationship matrix (NRM); A_22_ is the inverse of the numerator relationship matrix for genotyped animals; and G indicates the genomic relationship matrix for genotyped animals.

Estimation of variance components was performed using ssGBLUP through average information restricted maximum likelihood (AIREML) method. All analyses performed in this study were done using the BLUPF90 software family [22]. The corrected phenotypes were calculated using PREDICTF90, whereas variance components and GEBV were obtained using AIREMLF90 and BLUPF90, respectively.

### 2.9. Accuracy of Genomic Prediction

The genomic prediction accuracies for GBLUP and ssGBLUP were evaluated using a five-fold cross-validation scheme composed of five subpopulations that were randomly divided into more or less equally sized groups. Among the subpopulation, only one remained as the validation set, and the remaining four subpopulations were considered as the reference population. Cross-validation (CV) was done five times with each subpopulation given a chance to be used as a validation set. Moreover, the five-fold CV was replicated five times, resulting in five averaged accuracies for each trait used in this study. Evaluation of genomic prediction accuracy was calculated using the weighted average correlation across five validation sets following the formula used by Serão et al. [23] (Equation (5)):

(5) $$Accuracy=\frac{\sum_{i=1}^{5}n_{i}r_{i}\left(GEBV,y_{C}\right)}{\frac{\sum_{i=1}^{5}ni}{\sqrt{h^{2}}}}$$

where *r_i_* (GEBV,*y_c_*) indicates the correlation of GEBV obtained using GBLUP and ssGBLUP with *y_c_* and *n_i_* indicates the number of animals for each validation set. In addition, the bias of genomic prediction was evaluated based on the regression of corrected phenotypes on GEBV. A regression coefficient of 1 indicates unbiasedness, and thus values closer to 1 are the most preferable.

## 3. Results and Discussion

### 3.1. Descriptive Statistics

The descriptive statistics for all traits belonging to three categories, including meat quality traits, sensory characteristics, and fatty-acid composition of Duroc meat, are presented in Table 1. The average measurements for traits related to meat quality were as follows: the mean values for meat color were 49.24 ± 6.20 (lightness), 14.38 ± 1.87 (redness), and 6.07 ± 1.67 (yellowness), whereas the mean percentage for moisture content (MC), fat content (FC), water holding capacity (WHC), and cooking loss (CL) were 73.49 ± 1.48, 3.47 ± 1.50, 74.54 ± 6.26 and 12.73 ± 3.07, respectively. Further, the average values for pH24h post-mortem and shear force were 5.78 ± 0.19 and 4.91 ± 1.63 kg.f, respectively. The mean value for sensory characteristics and fatty-acid composition ranged from 5.04 to 5.17, and 0.54 to 42.81 percent with standard deviation (SD) ranging from 0.58 to 1.08, and 0.22 to 2.50, respectively.

### 3.2. Genetic Parameters

The genetic parameters with standard error estimated using ssGBLUP for meat quality traits, sensory characteristics, and fatty-acid composition of Duroc meat are presented in Table 2. The obtained heritability estimates for lightness (L*), redness (a*), and yellowness (b*) in this study were 0.45 ± 0.06, 0.72 ± 0.06, and 0.64 ± 0.06, respectively. The observed heritability for L* in this study was close with those observed by Willson et al. [24] and fell within the range (0.15 to 0.57) of 29 studies reviewed by Ciobanu et al. [25]. Contrastingly, a moderate to high heritability was observed by Gjerlaug-Enger et al. [26] for L* (0.28), a* (0.43), and b* (0.33) in Duroc meats. Moreover, Lee et al. [27] observed lower heritability estimates for L*(0.19), a* (0.36), and b* (0.28) for similar traits in Korean Berkshire.

Similarly, a moderate heritability estimate of 0.30±0.06 was observed for pH level at 24 h post-mortem (pH24h). This observation was similar to the heritability observed by Willson et al. [24] in the loin pH of Duroc pigs (0.39 ± 0.07) and fell within the heritability range of 0.07 to 0.39 from 33 studies reviewed by Ciobanu et al. [25] for ultimate post-mortem pH. Conversely, Suzuki et al. [28] and Lee et al. [27] reported lower heritability for pH24h of 0.07 ± 0.02 (Duroc) and 0.15 ± 0.05 (Berkshire), respectively.

In this study, the moisture content (MC), fat content (FC), water-holding capacity (WHC), and cooking loss (CL) presented moderate heritability estimates ranging from 0.25 to 0.30 with a similar standard error of 0.06, whereas a slightly low heritability of 0.19 ± 0.06 was observed for shear force (SF). All estimates except for WHC were lower than the findings of Ishii et al. [29] on Duroc pigs, with heritability estimates of 0.46, 0.52, 0.39, and 0.63 for MC, FC (intramuscular fat), CL, and SF, respectively, whereas a comparable estimate of 0.21 was observed for WHC.

For sensory characteristics, all traits were observed to have low heritability ranging from 0.08 to 0.14, with subjective meat color having the lowest heritability, while tenderness and palatability presented the highest. Although there have been limited studies about the heritability of pork sensory characteristics, the following literature still supports our findings. In a study by Lei et al. [30], equally low heritability values were obtained for sensory characteristics, including texture, flavor, juiciness, and palatability (overall opinion), with estimates ranging from 0.02 to 0.13. Similarly, Schwab and Baas [31] reported low heritability estimates of 0.12, 0.19, and 0.09 for flavor, tenderness, and juiciness, respectively, but obtained a higher heritability of 0.30 for subjective meat color. Such low observed heritability for sensory traits indicates a low response once included in the selection index. However, according to the Lei et al. [30], sensory traits gradually improved by selecting animals with intermediate pH and high IMF (intramuscular fat), since both of these traits were claimed to be positively correlated (genetically) to sensory traits. The initially claimed positive correlation of pH24h and IMF to sensory traits was verified through genetic and phenotypic correlation presented in Supplementary File, Table S2. The genetic correlation between fat content (FC) and sensory traits was positive, specifically for color (0.35 ± 0.42), flavor (0.31 ± 0.25), tenderness (0.25 ± 0.28), juiciness (0.26 ± 0.32), and palatability (0.31 ± 0.29). Meanwhile, a negative genetic correlation between pH24h and sensory traits was observed in this study, which was contrary to those observed by Lei et al. [30]. However, due to high standard error of genetic correlation in this study, careful interpretation of results is needed.

Furthermore, among the five fatty acids analyzed in this study, stearic, oleic, linoleic, and linolenic acids were highly heritable, with only palmitic acid found to be moderately heritable. Specifically, the obtained heritability estimates for the fatty acids considered in this study ranged from 0.27 to 0.50, with standard errors ranging from 0.06 to 0.07. Palmitic fatty acid recorded the lowest heritability of 0.27 ± 0.07, while oleic fatty acid presented the highest heritability of 0.50 ± 0.07. Fernandez et al. [32] reported comparable heritability for palmitic (0.31 ± 0.04), while lower estimates were observed for linoleic (0.29 ± 0.04), oleic (0.30 ± 0.03) and, stearic (0.41 ± 0.04) fatty acids measured using near-infrared spectroscopy (NIRS) in the longissimus dorsi muscle of Iberian pigs. Using similar technology (NIRS), Gjerlaug-Enger et al. [33] observed higher estimates for palmitic (0.51), stearic (0.54), oleic (0.57), and linoleic (0.46) fatty acids as well as lower heritability for α-linolenic fatty acid (0.25) in subcutaneous fat of Duroc pigs. According to Cameron et al. [34], intramuscular fat characteristics are more strongly influenced by nutritional effects than genetic effects, as nutritional methods offer an effective way of reducing the n-6:n-3 fatty acid ratio from pig meat. Although these traits are highly influenced by nutrition factors, our findings still indicate that the inclusion of fatty acids in the breeding program may further improve the meat quality of pork once combined with nutritional strategies.

Overall, the observed differences among heritability estimates for various traits might be attributed to various factors such as differences in breed and number of animals used, variations in animal models and fixed effects, large and unexplainable residual variances, differences in methodologies such as meat tissue sampling site and duration from slaughter to actual dissection, fatty acid analysis using different techniques such as NIRS and gas chromatography, and differences in the statistical method used to obtain the heritability estimates for each trait. Specifically, a marker-based heritability was obtained in this study using the ssGBLUP method. Despite obvious differences, our findings still demonstrate that the majority of traits associated with meat quality and fatty-acid composition of Duroc meat exhibited a moderate to high response to selection, whereas the traits related to sensory characteristics indicated limited genetic improvement once included in the breeding program.

### 3.3. Accuracy of Genomic Prediction Using GBLUP and ssGBLUP Methods

**Figure 1:**
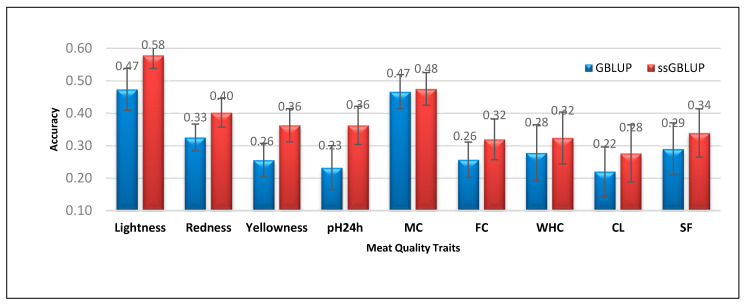
Prediction accuracies and standard errors obtained using genomic best linear unbiased prediction (GBLUP) and single-step GBLUP (ssGBLUP) for meat quality traits. pH24h—pH at 24 h post-mortem; MC—moisture content; FC—fat content; WHC—water-holding capacity; CL—cooking loss; SF—shear force.

**Figure 2:**
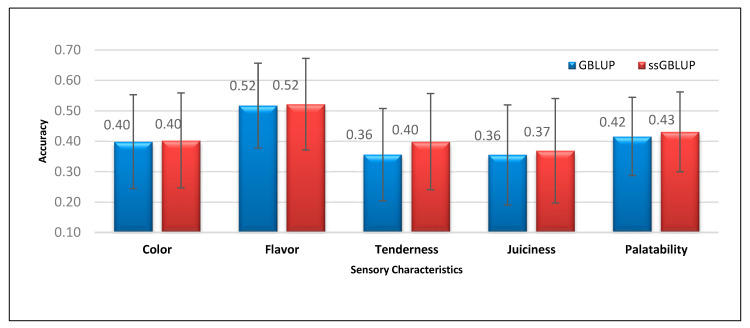
Prediction accuracies and standard errors obtained using GBLUP and ssGBLUP for sensory traits, including color, flavor, tenderness, juiciness, and palatability.

**Figure 3:**
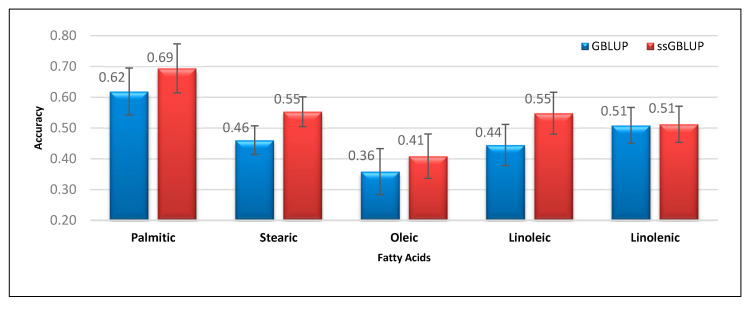
Prediction accuracies and standard errors obtained using GBLUP and ssGBLUP for palmitic, stearic, oleic, linoleic, and linolenic acids.

In this section, the genomic prediction accuracies of GBLUP and ssGBLUP for all traits related to meat quality traits, sensory characteristics, and fatty acid content were validated following the five-fold cross-validation scheme. The average genomic prediction accuracy for each trait and method are presented in Figure 1, Figure 2 and Figure 3. Figure 1 shows the genomic prediction accuracies and standard errors obtained using the GBLUP and ssGBLUP methods for meat quality traits, including meat color, pH24h, MC, FC, WHC, CL, and SF. For all meat quality traits, ssGBLUP obtained higher genomic prediction accuracy and comparable standard error when compared to the obtained values using GBLUP. For meat color traits, ssGBLUP exhibited 22.20%, 23.49%, and 42.02% higher genomic prediction accuracies than GBLUP for L*, a*, and b*, respectively. For pH24h, ssGBLUP also showed 56.07% higher genomic prediction accuracy than GBLUP. Similarly, ssGBLUP recorded 1.90%, 24.41%, 16.61%, 25.49%, and 16.93% higher genomic prediction accuracies than GBLUP for MC, FC, WHC, CL, and SF, respectively.

Figure 2 presents the prediction accuracies and standard errors for sensory characteristics of Duroc meat obtained using GBLUP and ssGBLUP. Unlike the percentage difference observed in meat quality traits, the majority of the traits related to sensory characteristics presented a lower advantage for ssGBLUP in terms of genomic prediction accuracy. Specifically, ssGBLUP showed only 1.03%, 0.88%, 11.85%, 3.77%, and 3.71% higher prediction accuracies than GBLUP for sensory characteristics, including subjective meat color, flavor, tenderness, juiciness, and palatability, respectively. In the case of meat toughness parameters like shear force and tenderness, the observed differences in their accuracy could be attributed to the lower heritability of the latter trait (0.14) than the earlier trait (0.19). Such observation is true in genomic prediction, since traits with low heritability gain more from the genomic information, especially when using a single-step method that includes all the pedigree, phenotype, and genotype information. Additionally, the majority of the prediction accuracy values for fatty-acid composition were higher in ssGBLUP than in GBLUP (Figure 3). For palmitic, stearic, oleic, linoleic, and linolenic fatty acids, ssGBLUP showed 12.17%, 20.16%, 13.91%, 23.21%, and 0.73% higher prediction accuracies than GBLUP for similar traits, respectively.

In general, ssGBLUP presented higher genomic prediction accuracy than GBLUP for all traits, with percentage difference ranging from 0.73% to 56.07%. Specifically, among the categories observed, meat quality traits presented the highest average percentage difference of 25.46%, followed by fatty acid content with 14.04%, and sensory characteristics, which recorded the least advantage for ssGBLUP of 4.25%. The better genomic prediction observed in this study can be mainly attributed to the larger reference population in ssGBLUP, since this method integrates the pedigree, phenotype, and genomic data. Similar to our findings, better genomic prediction accuracy for ssGBLUP than multi-step methods like GBLUP was observed in the study of Christensen et al. [13]. Meanwhile, the low percentage increase in the genomic prediction accuracy of ssGBLUP for sensory characteristics can be strongly attributed to the low heritability estimates observed for all traits in this category. This was supported by Gowane et al. [35], who concluded that models with higher heritability estimates will provide more accurate and less biased genomic predictions than those with low heritability.

### 3.4. Unbiasedness of Genomic Prediction Using GBLUP and ssGBLUP Methods

Apart from the prediction accuracy gained in the five-fold cross-validation, the level of unbiasedness for both statistical methods was obtained using linear regression analysis. The most acceptable regression coefficient for unbiased prediction should not deviate significantly from 1. Further, a regression coefficient less or greater than 1 indicates overestimation and underestimation, respectively. Therefore, the observed regression coefficient of less and greater than 1 in this study indicates overestimation or underestimation of the predicted breeding values against the true breeding values, respectively.

**Figure 4:**
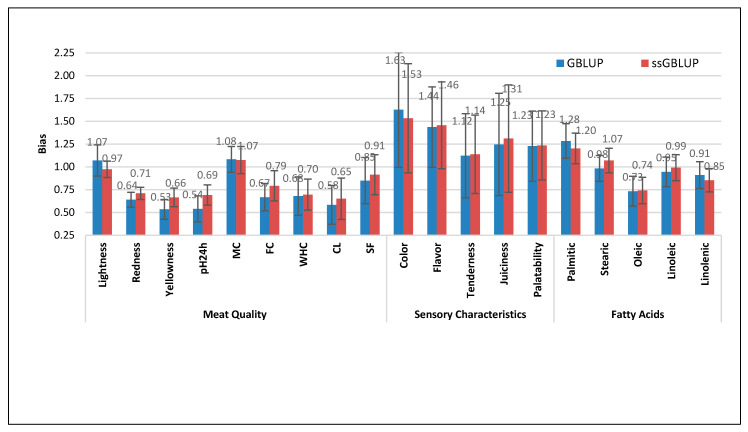
Bias estimates and standard error obtained using GBLUP and ssGBLUP for meat quality traits, sensory characteristics, and fatty-acid composition of Duroc meat. pH24h—pH at 24 h post-mortem; MC—moisture content; FC—fat content; WHC—water-holding capacity; CL—cooking loss; SF—shear force.

The regression coefficients for all traits in this study are shown in Figure 4. The ssGBLUP showed a regression coefficient of less than 1 for the majority of traits related to meat quality (ranging from 0.65 to 0.97). Cooking loss and lightness recorded the most and least biased estimates, respectively, whereas only moisture content presented slightly underestimated bias estimates of 1.07 ± 0.07. Meanwhile, more biased estimates were observed for a similar category using GBLUP with a regression coefficient ranging from 0.53 to 0.85; meat redness and shear force showed the most and least biased estimates, respectively, whereas lightness (1.07 ± 0.17) and moisture content (1.08 ± 0.14) presented marginally underestimated bias estimates.

Similar to meat quality traits, ssGBLUP obtained less bias estimates of 0.74 ± 0.14 and 0.99 ± 0.14 for oleic and linoleic fatty acids, respectively, compared to estimates of 0.73 ± 0.16 and 0.95 ± 0.16 obtained using GBLUP for similar traits. However, opposite findings were observed for linolenic and stearic fatty acids, as GBLUP provided less bias estimates of 0.91 ± 0.15 and 0.98 ± 0.14, respectively, compared to ssGBLUP bias estimates of 0.85 ± 0.13 and 1.07 ± 0.14 for similar traits. Moreover, moderate underestimated bias estimates were observed for palmitic fatty acid using both statistical methods.

Similar to moisture content and palmitic fatty acid, all traits related to sensory characteristics presented moderate to high underestimated bias estimates (Figure 4) using both statistical methods. Specifically, ssGBLUP recorded bias estimates ranging from 1.14 ± 0.43 to 1.53 ± 0.60. Similar findings were observed using the GBLUP method, with bias estimates ranging from 1.12 ± 0.46 to 1.63 ± 0.63. Further, both statistical methods observed that tenderness and subjective meat color obtained the least and most underestimated bias estimates among sensory characteristics of Duroc meat, respectively. These observed moderate to high underestimated bias estimates for sensory characteristics can be attributed to low heritability estimates for all traits in this category. According to Macedo et al. [36], the linear regression method can provide correct estimates of bias, slope, and accuracies provided that correct evaluation model is used, while better results are expected for traits with higher heritability estimates. Moreover, the same researchers concluded that evaluation model with incorrect heritability or hidden trends like environmental trends could provide a correct estimate of bias in terms of direction but with incorrect magnitude [36].

Despite the observed overestimation and underestimation of the majority of traits in this study, our findings still reveal that ssGBLUP provided less bias estimates than GBLUP. Specifically, the ssGBLUP recorded a less biased estimate for more than 60% of traits included in this study. Therefore, these findings indicate that the combination of almost two-fold phenotypes over GBLUP, pedigree, and genomic relationship matrix in ssGBLUP may replace the GBLUP method, which subsequently improves genomic prediction accuracy for breeding values of Duroc pigs in terms of meat quality traits, sensory characteristics, and fatty-acid composition.

## 4. Conclusions

The results of the cross-validation and regression analysis revealed that ssGBLUP obtained higher genomic prediction accuracy and less bias estimates than GBLUP for the majority of traits related to meat quality traits, sensory characteristics, and fatty-acid composition of Duroc meat. Furthermore, the high heritability estimates observed for meat quality traits and fatty-acid composition exhibited a moderate to high response to selection, whereas the low heritability estimates observed for sensory characteristics indicated a very slow response to selection.

## Figures and Tables

**Table 1:** Descriptive statistics for meat quality, sensory characteristics, and fatty-acid composition of Duroc meat.

Category/Trait	N	Mean	Min	Max	SD
End Weight	1237	97.25	75	136	8.78
**Meat Quality**					
Lightness	1145	49.24	34.25	66.95	6.20
Redness	1144	14.38	9.21	18.91	1.87
Yellowness	1145	6.07	2.28	12.74	1.67
pH24h	1224	5.78	5.23	6.83	0.19
MC (%)	1145	73.49	67.33	79.33	1.48
FC (%)	1123	3.47	0.30	14.02	1.50
WHC (%)	1145	74.54	54.57	94.08	6.26
CL (%)	1145	12.73	1.01	25.76	3.07
SF (kg.f)	1145	4.91	1.39	12.94	1.63
**Sensory**					
Color	1144	5.15	2.50	8.25	0.58
Flavor	1145	5.11	1.25	8.25	0.76
Tenderness	1145	5.17	1.25	8.75	1.08
Juiciness	1145	5.08	1.25	9.75	1.02
Palatability	1145	5.04	1.25	8.5	1.06
**Fatty Acids**					
Palmitic (C16:0, %)	1110	25.01	20.54	36.33	1.24
Stearic (C18:0, %)	1110	12.33	8.02	16.93	1.16
Oleic (C18:1, %)	1110	42.81	31.69	51.57	2.50
Linoleic (C18:2, %)	1109	8.53	4.61	14.98	1.75
Linolenic (C18:3, %)	1000	0.54	0.03	1.38	0.22

pH24h—pH level at 24 h post-mortem; MC—moisture content; FC—fat content; WHC—water holding capacity; CL—cooking loss; SF—shear force.

**Table 2:** Additive genetic variance (**σ^2^_a_**), residual variance (**σ^2^_e_**), phenotypic variance (**σ^2^_p_**), and heritability estimates (**h^2^**) with standard error for meat quality traits, sensory characteristics, and fatty-acid composition of Duroc meat.

Category/Trait	σ^2^_a_	σ^2^_e_	σ^2^_p_	h^2^
**Meat Quality**				
Lightness	4.08 ± 0.69	5.02 ± 0.51	9.10 ± 0.43	0.45 ± 0.06
Redness	1.42 ± 0.17	0.56 ± 0.10	1.98 ± 0.10	0.72 ± 0.06
Yellowness	0.88 ± 0.11	0.50 ± 0.07	1.38 ± 0.07	0.64 ± 0.06
pH24h	0.01 ± 0.002	0.02 ± 0.002	0.03 ± 0.01	0.30 ± 0.06
MC (%)	0.58 ± 0.13	1.35 ± 0.11	1.93 ± 0.09	0.30 ± 0.06
FC (%)	0.44 ± 0.12	1.32 ± 0.11	1.76 ± 0.08	0.25 ± 0.06
WHC (%0	8.91 ± 2.42	26.52 ± 2.16	35.43 ± 1.60	0.25 ± 0.06
CL (%)	1.93 ± 0.47	4.86 ± 0.41	6.79 ± 0.31	0.28 ± 0.06
SF (kg.f)	0.29 ± 0.10	1.26 ± 0.10	1.55 ± 0.07	0.19 ± 0.06
**Sensory**				
Color	0.02 ± 0.02	0.28 ± 0.02	0.31 ± 0.013	0.08 ± 0.05
Flavor	0.07 ± 0.03	0.50 ± 0.03	0.58 ± 0.02	0.12 ± 0.05
Tenderness	0.10 ± 0.07	0.98 ± 0.07	1.14 ± 0.05	0.14 ± 0.06
Juiciness	0.09 ± 0.05	0.91 ± 0.06	1.00 ± 0.04	0.09 ± 0.05
Palatability	0.02 ± 0.06	0.94 ± 0.07	1.10 ± 0.05	0.14 ± 0.06
**Fatty Acids**				
Palmitic (C16:0)	0.32 ± 0.09	0.88 ± 0.08	1.20 ± 0.06	0.27 ± 0.07
Stearic (C18:0)	0.54 ± 0.09	0.60 ± 0.07	1.13 ± 0.06	0.47 ± 0.07
Oleic (C18:1)	1.93 ± 0.31	1.96 ± 0.22	3.89 ± 0.19	0.50 ± 0.07
Linoleic (C18:2)	0.92 ± 0.17	1.39 ± 0.13	2.31 ± 0.11	0.40 ± 0.06
Linolenic (C18:3)	0.003 ± 6.8 × 10^−4^	0.005 ± 5.3 × 10^−4^	0.009 ± 4.2 × 10^−4^	0.41 ± 0.07

pH24h—pH level at 24 h post-mortem; MC—moisture content; FC—fat content; WHC—water holding capacity; CL—cooking loss; SF—shear force.

## Supplementary Materials

The following are available online at https://www.mdpi.com/2073-4425/11/9/1062/s1. Table S1. Genetic and phenotypic correlation among meat quality traits. Table S2. Genetic and phenotypic correlation between meat quality traits and sensory characteristics.
