# Supplementary material for: Single-Step Genomic Evaluation for Meat Quality Traits, Sensory Characteristics, and Fatty-Acid Composition in Duroc Pigs
Source: Genes (Basel). 2020 Sep 9;11(9):1062. doi: 10.3390/genes11091062 (PMC7563502; doi:10.3390/genes11091062)
Supplement: Supplementary file 1 [file genes-11-01062-s001.pdf]

Table S1. Genetic and phenotypic correlation among meat quality traits.

Traits	L*	a*	b*	pH24h	MC	FC	WHC	CL	SF
**L***		0.54 ± 0.09	0.50 ± 0.08	−0.05 ± 0.15	−0.23 ± 0.14	0.02 ± 0.16	−0.02 ± 0.15	−0.21 ± 0.16	−0.38 ± 0.24
**a***	0.25 ± 0.03		0.45 ± 0.08	0.18 ± 0.13	0.22 ± 0.13	−0.04 ± 0.14	0.41 ± 0.12	−0.36 ± 0.13	0.003 ± 0.17
**b***	0.48 ± 0.03	0.33 ± 0.03		0.10 ± 0.13	0.004 ± 0.13	0.14 ± 0.14	−0.06 ± 0.13	−0.04 ± 0.14	0.08 ± 0.19
**pH24h**	−0.10 ± 0.03	0.01 ± 0.03	−0.04 ± 0.03		0.1 ± 0.18	−0.14 ± 0.20	0.5 ± 0.16	−0.13 ± 0.19	0.56 ± 0.35
**MC**	−0.07 ± 0.03	0.09 ± 0.03	−0.12 ± 0.03	0.03 ± 0.03		−0.17 ± 0.19	0.09 ± 0.18	0.24 ± 0.19	0.53 ± 0.25
**FC**	0.11 ± 0.03	−0.08 ± 0.03	0.15 ± 0.03	0.01 ± 0.03	−0.22 ± 0.03		0.18 ± 0.19	0.09 ± 0.21	−0.45 ± 0.26
**WHC**	−0.19 ± 0.03	0.10 ± 0.03	−0.16 ± 0.03	0.28 ± 0.03	0.10 ± 0.03	0.00 ± 0.03		−0.21 ± 0.19	0.05 ± 0.54
**CL**	0.02 ± 0.03	−0.08 ± 0.03	−0.04 ± 0.03	−0.14 ± 0.03	0.07 ± 0.03	−0.04 ± 0.03	−0.22 ± 0.03		0.19 ± 0.86
**SF**	−0.07 ± 0.03	−0.07 ± 0.03	−0.005 ± 0.03	−0.08 ± 0.03	0.06 ± 0.03	−0.08 ± 0.03	−0.17 ± 0.03	0.02 ± 0.03	

Above diagonal, genetic correlation; below diagonal, phenotypic correlation. L*—Lightness; a*—redness; b*—yellowness; pH24h—pH value of meat after 24 hours post-mortem; MC—moisture content; FC—fat content; WHC—water holding capacity; CL—cooking loss; SF—shear force value.

Table S2. Genetic and phenotypic correlation between meat quality traits and sensory characteristics.

Traits	Color		Flavor		Tenderness		Juiciness		Palatability	
	r_g	r_p	r_g	r_p	r_g	r_p	r_g	r_p	r_g	r_p
**L***	0.53 ± 0.41	−0.01 ± 0.03	0.39 ± 0.25	0.02 ± 0.03	0.33 ± 0.29	0.02 ± 0.03	0.36 ± 0.31	0.01 ± 0.03	0.35 ± 0.26	0.03 ± 0.03
**a***	−0.08 ± 0.24	0.03 ± 0.03	−0.06 ± 0.18	0.04 ± 0.03	0.20 ± 0.21	0.02 ± 0.03	0.19 ± 0.24	0.05 ± 0.03	0.12 ± 0.18	0.05 ± 0.03
**b***	0.18 ± 0.25	−0.02 ± 0.03	0.09 ± 0.18	−0.01 ± 0.03	−0.18 ± 0.19	−0.05 ± 0.03	−0.16 ± 0.24	−0.06 ± 0.03	−0.08 ± 0.20	−0.04 ± 0.03
**pH24h**	−0.39 ± 0.47	0.06 ± 0.03	−0.36 ± 0.64	0.06 ± 0.03	−0.65 ± 0.40	0.12 ± 0.03	−0.38 ± 0.43	0.15 ± 0.03	−0.34 ± 0.37	0.13 ± 0.03
**MC**	−0.34 ± 0.34	−0.02 ± 0.03	−0.08 ± 0.23	−0.04 ± 0.03	−0.09 ± 0.30	−0.04 ± 0.03	−0.31 ± 0.30	−0.04 ± 0.03	−0.16 ± 0.26	−0.04 ± 0.03
**FC**	0.35 ± 0.42	0.07 ± 0.03	0.31 ± 0.25	0.09 ± 0.03	0.25 ± 0.28	0.09 ± 0.03	0.26 ± 0.32	0.09 ± 0.03	0.31 ± 0.29	0.11 ± 0.03
**WHC**	−0.33 ± 0.57	0.09 ± 0.03	−0.17 ± 0.28	0.12 ± 0.03	0.26 ± 0.25	0.22 ± 0.03	0.38 ± 0.28	0.22 ± 0.03	0.27 ± 0.24	0.21 ± 0.03
**CL**	0.16 ± 0.38	−0.10 ± 0.03	0.02 ± 0.26	−0.10 ± 0.03	−0.05 ± 0.28	−0.17 ± 0.03	−0.32 ± 0.29	−0.19 ± 0.03	−0.12 ± 0.26	−0.17 ± 0.03
**SF**	−0.60 ± 0.55	−0.16 ± 0.03	−0.67 ± 0.30	−0.18 ± 0.03	−0.73 ± 0.33	−0.34 ± 0.03	−0.89 ± 1.36	−0.24 ± 0.03	−0.67 ± 0.38	−0.31 ± 0.03

L*—Lightness; a*—redness; b*—yellowness; pH24h—pH value of meat after 24 hours post-mortem; MC—moisture content; FC—fat content; WHC—water holding capacity; CL—cooking loss; SF—shear force value.
